# Cerebral torque is human specific and unrelated to brain size

**DOI:** 10.1007/s00429-018-01818-0

**Published:** 2019-01-11

**Authors:** Li Xiang, Timothy Crow, Neil Roberts

**Affiliations:** 1 Edinburgh Imaging, School of Clinical Sciences, University of Edinburgh, Edinburgh, Scotland EH16 4TJ UK; 2 University Department of Psychiatry, Warneford Hospital, Oxford, England OX3 7JX UK

**Keywords:** Cerebral torque, Asymmetry, Speciation, Chimpanzee, Magnetic Resonance Imaging (MRI)

## Abstract

The term “cerebral torque” refers to opposing right–left asymmetries of frontal and parieto-occipital regions. These are assumed to derive from a lateralized gradient of embryological development of the human brain. To establish the timing of its evolution, we computed and compared the torque, in terms of three principal features, namely petalia, shift, and bending of the inter-hemispheric fissure as well as the inter-hemispheric asymmetry of brain length, height and width for in vivo Magnetic Resonance Imaging (MRI) scans of 91 human and 78 chimpanzee brains. We found that the cerebral torque is specific to the human brain and that its magnitude is independent of brain size and that it comprises features that are inter-related. These findings are consistent with the concept that a “punctuational” genetic change of relatively large effect introduced lateralization in the hominid lineage. The existence of the cerebral torque remains an unsolved mystery and the present study provides further support for this most prominent structural brain asymmetry being specific to the human brain. Establishing the genetic origins of the torque may, therefore, have relevance for a better understanding on human evolution, the organisation of the human brain, and, perhaps, also aspects of the neural basis of language.

## Introduction

What neural structure has allowed humans to exceed other animals in “cognitive ability”? Two hypotheses have been proposed. First, relative to body size, the human brain is disproportionally larger than the brains of other primates (Rilling 2006; Striedter 2005). Second, whilst the total volume and surface area of the left and right cerebral hemispheres are highly conserved, the human brain typically exhibits an asymmetry in shape that is often referred to as a counter-clockwise torque (Toga and Thompson 2003). The shape asymmetry is proposed as a potential substrate for hemispheric specialization of function, including left hemisphere dominance for language. There have, however, been many reports of asymmetries in the chimpanzee brain; for example, in Broca’s (Cantalupo and Hopkins 2001), Wernicke’s (Gannon et al. 1998) and other areas (Gilissen and Hopkins 2013; Hopkins 2013) and which challenge the second view. Some authors suggested that chimpanzees share the patterns of asymmetry with humans, though to a lesser degree (Gomez-Robles et al. 2013). However, this classical Darwinian concept of gradual transitions over long periods of time cannot explain the gap in functional abilities, particularly relating to language and handedness between humans and chimpanzees. These issues require further investigation using state-of-the-art image analysis techniques.

At the level of individual brain structures, many studies have reported finding a significant asymmetry of the planum temporale in the human brain, with a prevalence ranging between 60 and 83% and a sizable magnitude, such that the left side is of the order one-third larger than the right side (Witelson and Kigar 1988), and which is also supported by micro-anatomical measures (Chance et al. 2006). In an influential study, Gannon et al. (1998) reported detecting a significant leftward asymmetry of the planum temporale in the chimpanzee brain using a technique in which pieces of thin plastic were cut to the size of this structure in fixed post-mortem brains. However, the asymmetric spacing of mini-columns that may account for the surface area asymmetry of planum temporale in human brains has been reported to be absent in chimpanzees (Buxhoeveden et al. 2001; Chance 2014). On the other hand, there is now little convincing evidence that the classic language region known as Broca’s area is asymmetric in either the human or the chimpanzee brain. In particular, based on a review of the literature, Witelson and Kigar (1988) concluded that “there is no evidence of a statistically larger left than right Broca’s region” in the human brains and which was supported by a more recent review of the literature by Keller et al. (2009a) who highlighted the large variation in the anatomical definitions and the inconsistency in methodology. These authors went on to perform a comparative study of Broca’s area in humans and Broca’s area homolog in chimpanzees using unbiased stereological techniques to measure volume on 3D MRI scans obtained in vivo using an identical protocol and did not detect a significant asymmetry for either species (Keller et al. 2009b). The absence of significant asymmetry of Broca’s area in chimpanzee brains has also been reported by Schenker et al. (2010) and Xiang et al. (2018), based on different measures. Thus, whether asymmetry is a principal difference between the human and chimpanzee brain is still to reach consensus. In the present study, we focus on the cerebral torque and add detailed measurements of three main features of the torque to the findings reported in Xiang et al. (2018).

With regard to the torque, an archetypical view of which is depicted in Fig. 2 of Toga and Thompson (2003). There are many reports of it being present in the human brain and which have used a wide range of measurement techniques on CT and MR images acquired both in vivo and in vitro (Bear et al. 1986; Watkins et al. 2001; Weinberger et al. 1982; Good et al. 2001; Barrick et al. 2005). The first study of the cerebral torque in non-human primates was by LeMay (1976). Based on the measurement from the photographs of 28 great ape brains (12 orangutans, 9 chimpanzees, and 7 gorillas), the authors reported that the width of the occipital pole was larger on the left in 11 brains and vice versa in 6 brains, and that the right occipital petalia was present in 11 ape brains and vice versa in two. In an in-vivo MRI study of 31 chimpanzees, Hopkins et al. (2008) applied the VBM technique to explore brain asymmetry and reported rightward asymmetries in the frontal region and leftward asymmetries in the posterior parietal and occipital lobes in this chimpanzee population, although, in the VBM approach, it is difficult to distinguish the asymmetry caused by a potential offset in the position of the cerebral hemispheres along the antero-posterior axis from the asymmetry in the left–right tissue distribution. In an analysis of landmarks on the CT scans of skull endocasts obtained for a sample of extant and fossil specimens including 89 hominins and 110 African great apes (including chimpanzees, gorillas, and bonobos), Balzeau et al. (2012) reported the existence of the petalia in great apes, and concluded that the petalia pattern is not human specific. However, inconsistent observation was reported by Holloway and De La Costelareymondie (1982). In a study of a large collection of 190 hominoid endocasts including specimens for 34 chimpanzees, 40 gorillas, and 41 bonobos, the authors did not find the torque to be present in great apes and concluded that “while true asymmetries of a cortical nature may exist in the extant pongids, their patterns and underlying evolutionary history have not been as strongly selected for”. In a recent in vivo MRI study, Xiang et al. (2018) performed a detailed analysis of 3D positional asymmetries of the surface of the human and chimpanzee brain. The torque and several notable local asymmetries, such as greater depth of the Superior Temporal Sulcus (STS) in the right cerebral hemisphere, were evident in the asymmetry maps of the human brain, but were not present in the chimpanzee brain.

In the present study, the analysis of Xiang et al. (2018) is extended to allow a more detailed study of brain torque from in vivo MRI scans of 91 human and 78 chimpanzee brains. Three aspects of the brain torque have been measured: (1) *petalia* whereby one cerebral hemisphere protrudes anteriorly and the other posteriorly in the antero-posterior direction, (2) *shift* whereby one cerebral hemisphere moves upwards anteriorly and downwards posteriorly relative to the other in the dorso-ventral direction, and (3) *bending* whereby the brain tissue in one hemisphere crosses the midline to displace tissue in the other hemisphere. We find that all three aspects of the torque are specific to the human brain and unrelated to brain size.

## Materials and methods

### Subjects

3D MRI brain scans of human subjects were acquired at the Edinburgh Imaging facility QMRI, University of Edinburgh, UK, and the Oxford Centre for Magnetic Resonance (OCMR), University of Oxford, UK. Approval was obtained separately at each site from the local Research Ethics Committee and subjects provided fully informed written consent prior to taking part. Altogether, 91 healthy subjects (mean age 33.5 years, 39 females, and 52 males) were included in the study. However, the handedness and the body weight of all the participants are not completely known. For the human subjects recruited in Edinburgh, MR images were obtained using a 3D MPRAGE sequence on a 3 T Verio MRI system (Siemens Medical Systems, Erlangen, Germany). Acquisition parameters were TR = 2300 ms, TE = 2.98 ms, TI = 900 ms, Flip angle = 9°, and FOV = 256 mm × 256 mm with an isotropic voxel resolution of 1 mm. For the human subjects recruited in Oxford, MR images were obtained using a 3D fast low-angle shot (FLASH) sequence on a Sonata 1.5 T MR system (Siemens Medical, Erlangen, Germany). Acquisition parameters were TR = 5400 ms, TE = 76 ms, Flip angle = 90°, 256 × 160 slice matrix comprising 208 contiguous slices with an isotropic voxel resolution of 1 mm. MRI scanning of the 78 chimpanzees (50 females and 28 males) which weighted an average of 64 kg was performed at Yerkes National Primate Research Centre (YNPRC) in Atlanta, Georgia, US. Chimpanzees were immobilized by ketamine injection (10 mg/kg) and subsequently anesthetized with propofol (40–60 mg/kg/h) before transportation to the MRI facility where they remained anesthetized (total time ~ 2 h) for the MR imaging and return to the home compound. Chimpanzees were scanned supine with a human head coil. T1-weighted magnetization-prepared rapid-acquisition gradient echo (MPRAGE) MR images were obtained using a Siemens 3 T Trio MR system. Acquisition parameters were TR = 2300 ms, TE = 4.4 ms, TI = 1100 ms, flip angle = 8, and FOV = 200 mm 9200 mm. The data matrix size was 320 × 320 with an isotropic voxel resolution of 0.6 mm.

### Image processing

All MR images were pre-processed in FSL (http://fsl.fmrib.ox.ac.uk/fsl/fslwiki/) including skull strip, bias field correction, and brain normalization using 7 degrees of freedom transformations (i.e., 3 translations, 3 rotations, and 1 uniform scaling) without distorting the morphological shape of brains, and were analyzed using the standard FreeSurfer processing pipeline (https://surfer.nmr.mgh.harvard.edu/), in which the surface-based module enables high-quality cerebral surface reconstruction from the brain volume data with subvoxel accuracy (Dale et al. 1999). The processing pipeline has been described in details elsewhere (Hopkins et al. 2017). Based on a method available in FreeSurfer (Schaer et al. 2008), the outer surface of the cerebrum was extracted from a smoothed cerebral hemisphere volume, on which the morphologic closing operation was applied to fill the sulci. The computation of brain dimensions and measurement of the cerebral torque was performed for this surface.

**Fig. 1:**
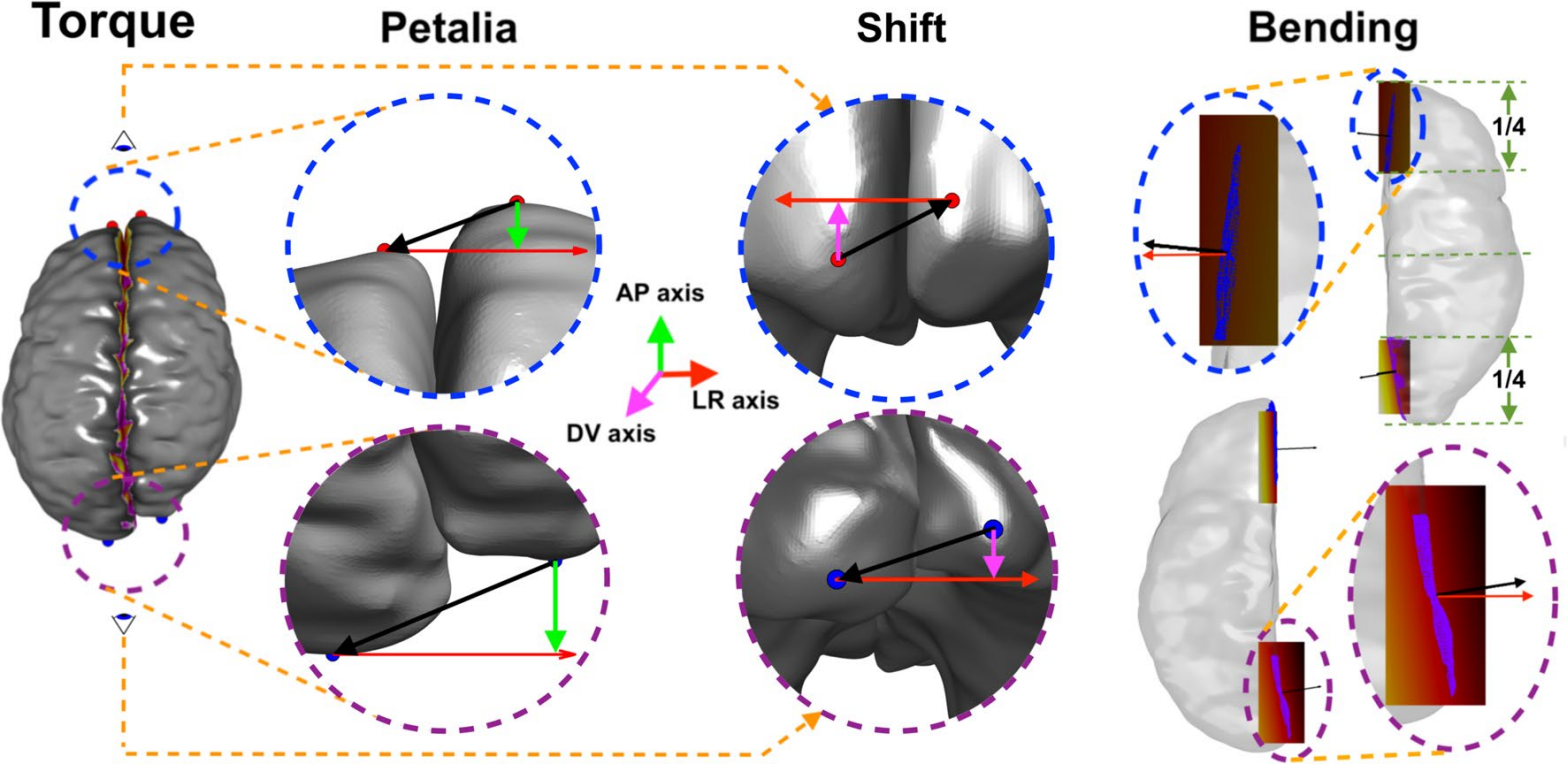
Illustration of brain torque computation. The frontal and occipital poles (highlighted in red and blue, respectively) were computed as the most extreme points on each cerebral hemisphere in the antero-posterior direction. The relative displacements (black arrow) of the left- and right-frontal, and occipital poles, in the antero-posterior, and dorso-ventral, directions correspond to petalia (column 2, green arrows), and shift (column 3, magenta arrows), respectively. For each cerebral hemisphere, the vertices of the medial surfaces of the cerebral hemispheres in the first (blue points) and last quarter (purple points) of the brain along the antero-posterior direction were used to fit 3D least-squares planes for the frontal and occipital regions (column 4), respectively. The frontal and occipital bending was measured as the angles between the *x*-axis (in red) and the normal of the fitted plane (in black), and was averaged between the two cerebral hemispheres

Despite the fact that the 3D MRI brain image has already been normalized to the MNI coordinate system in FSL, the low-dimensional linear registration used in the normalization step is often insufficient to align the inter-hemispheric fissure to the *x* = 0 plane that is commonly considered as the midline plane of the brain (Good et al. 2001; Kennedy et al. 1999; Lyttelton et al. 2009; Watkins et al. 2001). The deviation of the two planes is likely to affect the computation of brain asymmetry as well as the brain dimensions. To improve the accuracy of the measurements, a middle-sagittal plane (MSP) was computed based on the central portion of the brain where deviation is minimal. To achieve this, the MSP was estimated as the plane that best fits the vertices on the medial surface of the brain. The brain orientation was refined by rotating the brain surface with an angle between the plane *x* = 0 and the estimated MSP [Fig. 1, column 1, a more detailed description was given in Xiang et al. (2018)].

The dimensions (i.e., antero-posterior length, dorso-ventral height, and latero-medial width) of the brain as a whole and of the individual cerebral hemispheres were measured as the dimensions of the smallest orthogonal parallelepiped (i.e., so-called “bounding-box”) that could be constructed to enclose the smooth outer surface of each cerebral hemisphere with edges parallel to the three axes of the MNI coordinate system. The uniform scaling factor previously computed with FSL was applied to correct the measured dimensions to the true dimensions in native space. The ratios of the linear measures were also computed (i.e., length/width and height/width). The dimensional asymmetries were defined as the difference (i.e., L–R) of the linear measurements of length, height and width between the left and right cerebral hemispheres.

The frontal and occipital poles were computed as the most extreme points on each cerebral hemisphere along the antero-posterior axis and the description petalia refers to the respective displacements of the left and right frontal and occipital poles along this axis (Fig. 1, column 2) and shift as the corresponding displacements along the dorso-ventral axis (Fig. 1, column 3). Bending was computed as the angles between the best-fitting plane and plane *x* = 0 in the frontal and occipital regions. The procedure can be sub-divided into the following steps: (1) compute the surface normal and the angle *θ* between *x*-axis and the estimated normal at each cerebral surface vertex, (2) identify the vertices that belong to the medial cerebral surface as those associated with angles *θ* less than 40° (vertices on the lateral cerebral surface normally have larger angles, i.e., *θ* > 40°). From these vertices, identify those in the first (frontal region) and last quarters (occipital region) of the brain along the antero-posterior direction and (3) compute the least-squares planes that best fit the points belonging to the frontal and occipital regions, respectively, and compute the frontal and occipital bending as the angles between the normal of individual planes and the *x*-axis in associated regions (Fig. 1, column 4).

### Statistical analysis

Multiple analysis of variance (MANOVA) was applied to determine potential species and sex differences in brain size. The brain dimensions (i.e., length, height, and width) are dependent variables and sex and species between group factors. For individual species, two-tailed one-sample *t* tests were applied to measure the asymmetry of each brain variable under the null hypothesis that the difference between the left and right hemispheres is equal to zero. The prevalence of the pattern of asymmetries was compared between species using a Chi-squared two-sample test. Findings were considered statistically significant at *p* < 0.01.

## Results

The linear measurements of the brain of both human and chimpanzee brains are presented in Table 1. Based on the linear measurement of the whole cerebral surface, the human brain is significantly longer (95% CI for the effect of species is 62–66 mm), higher (95% CI is 41–44 mm), and wider (95% CI is 45–48 mm) than that of the chimpanzee brain by factors of 1.57, 1.57 and 1.54, respectively. Within each species, the ratio of length, height, and width is 1.31:0.88:1 in humans and 1.28:0.86:1 in chimpanzees, respectively. MANOVA [*F*(2,166) = 4.96, *p* = 0.008] based on the ratios between length and height to width and subsequent ANOVA confirmed relatively greater expansion in the antero-posterior [*F*(1,167) = 9.79, *p* = 0.002] and dorso-ventral [*F*(1,167) = 5.47, *p* = 0.02] directions compared to the lateral–medial direction in humans. Sex effects were present in both humans [*F*(3,87) = 24.22, *p* < 0.0005] and chimpanzees [*F*(3,74) = 7.16, *p* < 0.0005], although the normalized difference between the sexes is comparatively smaller in the chimpanzee than in the human brain.

**Table 1:** Brain dimensions and ratios

(mm)	Left hemisphere			Right hemisphere			Whole cerebral surface				
	Length	Height	Width	Length	Height	Width	Length	Height	Width	Length/Width	Height/Width
Humans											
All	173.8 ± 7.5	116.0 ± 5.2	70.3 ± 3.2	172.9 ± 7.4	116.8 ± 5.1	70.0 ± 3.5	174.9 ± 7.6	117.7 ± 5.0	134.0 ± 5.6	1.31	0.88
Male	177.0 ± 7.2	117.8 ± 4.9	71.9 ± 2.9	176.1 ± 7.0	118.5 ± 5.1	71.6 ± 2.9	178.2 ± 7.1	119.5 ± 4.7	137.1 ± 4.6	1.30	0.87
Female	169.6 ± 5.6	113.7 ± 4.7	68.1 ± 2.2	168.7 ± 5.6	114.5 ± 4.3	67.4 ± 2.6	170.5 ± 5.7	115.3 ± 4.2	129.9 ± 3.9	1.31	0.89
Chimpanzees											
All	110.4 ± 4.2	74.5 ± 3.6	45.1 ± 2.1	110.4 ± 4.2	74.4 ± 3.8	45.1 ± 2.1	111.1 ± 4.1	75.1 ± 3.7	87.2 ± 3.8	1.28	0.86
Male	112.6 ± 4.5	75.8 ± 3.9	45.9 ± 1.9	112.4 ± 4.5	75.8 ± 4.1	45.8 ± 1.7	113.3 ± 4.4	76.4 ± 3.9	88.6 ± 3.3	1.28	0.86
Female	109.2 ± 3.5	73.8 ± 3.3	44.6 ± 2.1	109.2 ± 3.6	73.6 ± 3.5	44.7 ± 2.2	109.9 ± 3.5	74.4 ± 3.4	86.4 ± 3.9	1.27	0.86

For each subject, the length, height and width of the whole brain and the two cerebral hemispheres were measured based on three bounding-boxes constructed independently for the relevant brain surfaces. For the human brain, the normalized difference in brain size between the sexes is between 3 and 5%, while, for the chimpanzee, the difference between the sexes is between 2 and 3%

The brain asymmetry measures are presented in Table 2 and the prevalence of the torque in each species can be found in Table 3. According to Table 2 (left panel**)**, the left hemisphere of the human brain was significantly longer [*t*(90) = 4.77, *p* < 0.0005] and less high [*t*(90) = − 3.30, *p* = 0.001] than the right hemisphere; however, there was no significant global width asymmetry. For chimpanzees, no significant asymmetry was present in any of the dimensions.

**Table 2:** Difference of brain measures between the left and right cerebral hemispheres were examined using one-sample two-tailed t tests

Asymmetry (L–R)				Torque					
(mm)	Length	Height	Width	Petalia		Shift		Bending	
				Anterior	Posterior	Anterior	Posterior	Anterior	Posterior
Humans									
AVG	0.92 ± 1.84	− 0.73 ± 2.11	0.45 ± 2.74	− 0.67	− 1.58	− 0.57	− 1.30	0.04	3.63
*t*(90)	4.77	− 3.30	1.56	− 4.94	− 7.69	− 0.89	− 2.66	0.22	6.65
*p* (two-tail)	< * 0.0005*	*0.001*	0.123	< * 0.0005*	< * 0.0005*	0.38	*0.01*	0.82	< * 0.0005*
Chimpanzees									
AVG	0.02 ± 1.38	0.11 ± 1.45	− 0.03 ± 1.11	− 0.18	− 0.20	− 0.77	0.25	− 0.35	− 0.39
*t*(77)	0.14	0.69	− 0.25	− 1.80	− 1.59	− 1.62	0.65	− 1.76	− 1.14
*p* (two-tail)	0.89	0.49	0.81	0.08	0.12	0.11	0.52	0.08	0.26

The aspects of cerebral asymmetry that proved to be significant with a significance level of 0.01 are highlighted in italics

**Table 3:** Prevalence of four configurations of frontal and occipital petalia/shift/bending (RF/LO, LF/RO, RF/RO, and LF/LO) in each species

	Petalia			Shift			Bending		
	Frontal			Frontal			Frontal		
	LF (%)	RF (%)		LF (%)	RF (%)		LF (%)	RF (%)	
*Human*									
Occipital									
LO	20.88	60.44	81.32%	23.08	39.56	62.64%	14.29	7.69	21.98%
RO	9.89	8.79	18.68%	20.88	16.48	37.36%	30.77	47.25	78.02%
	30.77	69.23		43.96	56.04		45.05	54.95	
*Chimpanzee*									
Occipital									
LO	21.79	30.77	52.56%	19.23	24.36	43.59%	23.08	34.62	57.69%
RO	17.95	29.49	47.44%	21.79	34.62	56.41%	16.67	25.64	42.31%
	39.74	60.26		41.03	58.97		39.74	60.26	

**Fig. 2:**
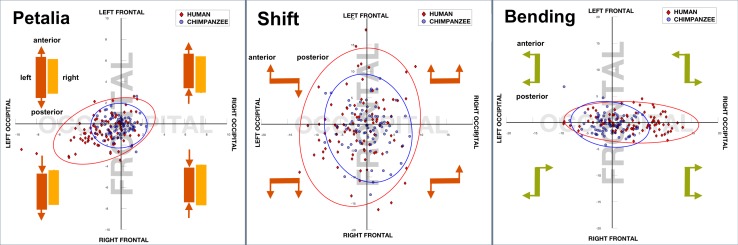
Cerebral torque in humans and chimpanzees. Plots of occipital (*x*-axis) and frontal (*y*-axis) petalia/shift/bending with 95% confidence ellipses are shown for humans (red diamonds) and chimpanzees (blue circles). In the case of petalia (left panel), the values for the majority of human subjects data are located in the left-bottom quadrant, indicating that the left hemisphere has an overall posterior shift compared to the right side. For shift (middle panel), human subjects demonstrate a modest but significant downward shift at the occipital pole in the left hemisphere compared to the right. For bending (right panel), human subjects show a directional rightward occipital bending, but there is no significant frontal bending. Values of all three measurements are randomly distributed in chimpanzees

Both the frontal and occipital petalia (see Table 2) were highly significant in humans [*t*(90) = − 4.94, *p* < 0.0005 and *t*(90) = − 7.69, *p* < 0.0005, respectively], whereas no significant asymmetry was found in either petalia in chimpanzees. According to the data distribution in Fig. 2, the combination of right-frontal and left-occipital petalia was most typical in humans (60%) consistent with the presence of the torque (Best 1988). In comparison, the configuration of the petalia was randomly distributed in chimpanzees (31%), which was significantly different to humans (Chi = 14.85, *p* < 0.0005). In addition, the left-occipital pole of the human brain was shifted significantly downward relative to the right [*t*(90) = − 2.66, *p* = 0.01], which was not the case in the chimpanzee brain. No significant relative shift can be found at the frontal pole in both species. With regard to bending, humans showed a significant rightward occipital bending *t*(90) = 6.65, *p* < 0.0005, which was not found in chimpanzees. By the Chi-squared test, the distribution of occipital bending differed significantly between humans and chimpanzees (Chi = 22.63, *p* < 0.0005). There was no significant sex effect found in asymmetries in either species.

Total intracranial volume (1.54 ± 0.23 dm^3^) computed using the FreeSurfer image analysis pipeline was significantly correlated with the product of length, height and width measures (*r* = 0.67, *p* < 0.0005). The correlations between asymmetries and this volume are, however, not significant (*r* = − 0.03, *p* = 0.77 in length, *r* = 0.02, *p* = 0.81 in height, *r* = − 0.02, *p* = 0.86 in width, *r* = − 0.08, *p* = 0.46 in frontal petalia, *r* = − 0.02, *p* = 0.83 in occipital petalia, *r* = 0.11, *p* = 0.29 in occipital bending, and *r* = 0.11, *p* = 0.28 in occipital shift). Pairwise Pearson’s correlations indicate that length asymmetry of the cerebral hemispheres in the human cohort is highly positively correlated with occipital petalia (*r* = 0.77, *p* < 0.0005) and negatively correlated with frontal petalia although with a lesser correction coefficient (*r* = − 0.25, *p* = 0.02). The occipital petalia was significantly more prominent than the frontal petalia *t*(90) = 4.77, *p* < 0.0005. In addition, a significant correlation was observed between length asymmetry and occipital bending (*r* = 0.57, *p* < 0.0005), and between occipital petalia and occipital bending (*r* = 0.63, *p* < 0.0005).

## Discussion

Two main findings distinguish the human from the chimpanzee brain: (1) larger brain size with preferential expansion in the antero-posterior and dorso-ventral axes relative to the left–right axis and (2) the torque whereby the right-frontal and left-occipital poles are more prominent (i.e., petalia) than the contralateral poles; the left-occipital pole is shifted downward (i.e., shift) compared to the right and bends towards the right side (i.e., bending) and dimensional asymmetries whereby the left hemisphere is elongated and with reduced height compared to the right. The asymmetries are independent of brain size. To our knowledge, this is the first study to systemically examine the torque, particularly the “shift” and “bending”, in living human and great ape brains as well as the relationship of asymmetries to linear brain size.

Most previous linear measures of the irregularly shaped brain have necessarily been obtained on arbitrarily defined 2D sections (Turkheimer 1989). In the present study, all the brain measures were computed from the 3D brain surface obtained in vivo from 91 humans and 78 chimpanzees using MRI. The linear measurements of the brain of humans and chimpanzees were found to be highly correlated with brain volume (*p* < 0.0005), in line with the proposition that linear brain dimensions are simple and reliable indices of brain size (Gomori et al. 1984; Hamano et al. 1993; Reinard et al. 2015) on an evolutionary timescale.

The average body mass of the captive chimpanzee subjects in this study is 64 ± 15 kg (much heavier than the body mass of wild chimpanzee reported in Pusey et al. 2004, i.e., 39.0 kg for males and 31.3 kg for females). The weight of the human subjects was unfortunately not recorded, however the average weight of the European population is 71 kg (Walpole et al. 2012). Thus, according to the linear measurements, the human brain is disproportionately large relative to the chimpanzee with regard to body weight. The phylogenetic increase in brain size is allometric. Greater growth was found in the antero-posterior direction, which is the principal direction of neuroanatomical diversity in the central nervous system (Best 1988; Gilles et al. 1983). The greater growth in this direction accords with relatively speedy prenatal (Sakai et al. 2012) and prolonged postnatal (Owen 1859) growth. Because greater brain size is associated with more neural tissue, and thus greater processing capacity, an increase of brain size has been considered as the basis of the emergence of human intelligence (Jerison 1973; Pilbeam and Gould 1974; Tramo et al. 1998), tool making (Ko 2016), and language (Lenneberg 1967b). However, uniform change in size alone cannot account for the specific human ability to acquire language as brain size is substantially reduced in nanocephalic dwarfism (Lenneberg 1967a; Seckel 1960) without consistent impairment of the capacity for language. Nor have elephants evolved a linguistic competence parallel to or exceeding our own; nevertheless, the singularity of the elephant’s trunk has been discussed as a parallel to human language in evolutionary theory (Pinker 1994).

The primary finding of the study is the uniqueness of asymmetries in the human brain. Petalia were found to be significant in both frontal and occipital regions (*p* < 0.01) in the human brain, indicating an overall posterior sheer of the left hemisphere compared to the right. The pattern of right-frontal and left-occipital protrusion appears in 60% of the human population, lying in the range of frequencies reported by two studies of endocasts, namely 79% by Holloway and De La Costelareymondie (1982) and 44% by Balzeau et al. (2012). In contrast to humans, the results relating to great apes have been inconsistent. Holloway and De La Costelareymondie (1982) reported a prevalence of petalia of 12% in great apes and a significantly different petalia patterns between humans and great apes was confirmed by Chi-square statistics. Balzeau et al. (2012) observed a higher prevalence of right-frontal and left-occipital pattern of petalia in great apes at 35% that is comparable with the cases in humans (44%) and significant left-occipital petalia in both great apes (*p* < 0.05) and humans (*p* < 0.01). On this basis, the authors concluded that this species shared a similar pattern of asymmetries with humans. In our study, the prevalence of the petalia pattern in chimpanzee is closer to the latter study at 31%. However, according to *t* tests of directional asymmetry, neither the right-frontal (*p* < 0.0005) or left-occipital (*p* < 0.0005) petalia found in human cohort was significant in the chimpanzee cohort (*p* > 0.05), in addition to which the Chi-square test for difference in prevalence between human and chimpanzee points to the same conclusion as drawn by Holloway and De La Costelareymondie (1982) and Zilles et al. (1996) that petalia are not present in chimpanzees on a population level. In addition to petalia, a significant downward shift (*p* = 0.01) and rightward bending (*p* < 0.0005) of the occipital lobes was found in the human brain but not in the chimpanzee brain. The lack of directional bending in chimpanzee brains is in line with a study by Hou et al. (2018), in which the unique occipital bending in humans was found to be associated with asymmetry in Sylvian Fissure that is also unique to humans. The elongation and reduced height of the left hemisphere observed in only human brains correlates with occipital petalia and bending, and is compatible with the posterior extension of the temporo-occipital region and the greater length of the Sylvian Fissure in the left hemisphere (Cunningham 1892; Rubens et al. 1976). In the previous studies, the torque is revealed by corresponding asymmetries in the skull (Balzeau and Gilissen 2010; Balzeau et al. 2012) or in gross volumetric measures of frontal and occipital regions (Watkins et al. 2001; Weinberger et al. 1982) without separating different features of the torque from each other (Chance et al. 2005). In this study, a comparison is directly made between 3D MRI scans obtained in vivo using an identical protocol for groups of humans and chimpanzees with reasonable sample size. The automated analysis procedures avoid the subjective judgment of an operator who has knowledge of the hypothesis being tested. Furthermore, tests of variables that have not previously been examined are included in the present study. In particular, detailed measurements have been obtained for three prominent features of the cerebral torque (i.e., petalia, shift, and bending).

The non-significant correlations between brain size and asymmetries suggest that the factors influencing brain size and cerebral asymmetry may have independent phylogenetic origins. Thus, the absence of asymmetry in the chimpanzee brain in the present study is not related to smaller brain size. Instead, the absence of asymmetry is interpreted as reflecting a difference in the phylogenetic history since the separation of the species. This is supported by reports, from analysis of photographs, of length asymmetry of fetal and newborn brains (LeMay 1976) and corticospinal tract asymmetry in fetal and neonatal autopsy material (Yakovlev and Rakic 1966).

The mechanisms underlying the development of brain asymmetry are not well understood. Two theories have been proposed. Some (Crow 1993, 1994) have hypothesized that brain asymmetry appeared as a late and “punctuational” step in the human phylogeny, initiated by a genetic mutation and is a potential anatomical correlate of language. Others however, have proposed that asymmetry is simply an organizing principle “providing for more efficient programming through the allocation of different functions to the two cerebral hemispheres” as a result of increasing brain size (Corballis 2010). The fact that cerebral asymmetry is absent from the brain of the chimpanzee and is un-correlated with brain size is more readily reconciled with a saltational, i.e., relatively abrupt genetic origin (Annett 1985), in the hominid lineage than as a cross-specific adaptation of generalized mammalian neuro-development.

The existence of torque is consistent with the lateralized neuro-embryologic development model proposed by Best (1988), in which maturation of the brain occurs along a 3D diagonal growth vector running from ventral right-frontal motor and primary sensory areas to dorsal left-posterior and tertiary association areas. The more striking asymmetry in the posterior region than the anterior is in line with Best’s (1988) prediction that “the gross morphologic effect of earlier-emerging right-frontal-motor regions may become attenuated by the later, left-biased growth of the tertiary association cortex in the prefrontal region”. The between species difference in cerebral torque also appears to be greater in the occipital lobe. The symmetry of the chimpanzee brain suggests a difference in neuro-development compared to humans. Although consideration of its specific effects on brain function and cognition are beyond the scope of the current study, the unique existence of torque in the human brain points to a correlate to the high-level cognitive ability of human being.

There are two main limitations of the study. Firstly, the lack of complete handedness information in human participants restricts exploration of the association between handedness and asymmetries which was previously reported in (Zilles et al. 1996; LeMay 1976). However, in a recent study, Kong et al. (2018) did not find a significant relationship between brain asymmetry and handedness in a large human cohort of 17,141 healthy individuals. Secondly, despite the major advantages of being able to compare 3D MRI scans obtained in vivo for cohorts of humans and chimpanzees, it is to be acknowledged that the published studies of endocasts refer to natural populations with a wide geographic range and greater sample sizes as compared to the captive Yerkes chimpanzee group. The findings of the present study are, however, in line with the observations by Holloway and De La Costelareymondie (1982) based on endocasts of great apes including gorilla, bonobo, and chimpanzees. Besides, it is also worth mentioning that the measurements reported in the present study are not identical to, for example, measurements obtained using manual techniques such as caliper width and length (LeMay 1976), planimetric approaches for Region of Interest (ROI) analyses (Hopkins et al. 1998; Hopkins and Marino 2000), computer-based techniques such as voxel-based morphometry (VBM) (Hopkins et al. 2008), or analysis of landmarks on CT scans of endocasts (Balzeau and Gilissen 2010; Balzeau et al. 2012). In future work, it will be interesting to compare the application of the different methods to obtain measurements for the same database and which will provide a more detailed description of the phenomenon referred to as the torque.

In conclusion, we have shown that cerebral torque and asymmetries in the dimensions of the cerebral hemispheres are specific human attributes that are independent of brain size, and perhaps, a consequence of a “macro-mutation” during human evolution (Annett 1985; Crow 1993, 1994) that led to the development of a three-dimensional maturation gradient in the embryonic brain (Best 1988).
